# Dog Wool Microparticles/Polyurethane Composite for Thermal Insulation

**DOI:** 10.3390/polym12051098

**Published:** 2020-05-11

**Authors:** Francisco Claudivan da Silva, Helena P. Felgueiras, Rasiah Ladchumananandasivam, José Ubiragi L. Mendes, Késia Karina de O. Souto Silva, Andrea Zille

**Affiliations:** 1 Post-graduate Program in Mechanical Engineering (PPGEM), Department of Mechanical Engineering, Federal University of Rio Grande do Norte, Natal 59078-970, Brazil; clauditextil@yahoo.com.br (F.C.d.S.); rlsivam@gmail.com (R.L.); ubiragi@ct.ufrn.br (J.U.L.M.); 2 Foundation for the Promotion of Research of the State of Rio Grande do Norte–FAPERN, Natal 59064-901, Brazil; 3 2C2T-Centro de Ciência e Tecnologia Têxtil, Universidade do Minho, Campus of Azurém, 4804-533 Guimarães, Portugal; helena.felgueiras@2c2t.uminho.pt; 4 Textile Engineering Department, Federal University of Rio Grande do Norte, Natal 59078-970, Brazil; kesiasouto@hotmail.com

**Keywords:** dog wool fibers, fillers, polyurethane, eco-composites, renewable resources

## Abstract

A polyurethane (PU)-based eco-composite foam was prepared using dog wool fibers as a filler. Fibers were acquired from pet shops and alkaline treated prior to use. The influence of their incorporation on the PU foams’ morphological, thermal, and mechanical properties was investigated. The random and disorganized presence of the microfibers along the foam influence their mechanical performance. Tensile and compression strengths were improved with the increased amount of dog wool microparticles on the eco-composites. The same occurred with the foams’ hydration capacity. The thermal capacity was also slightly enhanced with the incorporation of the fillers. The fillers also increased the thermal stability of the foams, reducing their dilatation with heating. The best structural stability was obtained using up to 120 °C with a maximum of 15% of filler. In the end, the dog wool waste was rationally valorized as a filler in PU foams, demonstrating its potential for insulation applications, with a low cost and minimal environmental impact.

## 1. Introduction

Global energy consumption is estimated to increase by 53% within the next 10 years [1]. One of the simplest and most cost-effective ways to reduce the energy demands and the greenhouse gas emissions is through building insulation. If properly selected, an effective insulation may save energy by requiring less for space cooling in the summer and heating in the winter, and thus reducing the use of natural resources (e.g., petroleum and gas) [2]. Thermal insulation is achieved by means of a material or composite materials endowed with high thermal resistance. Over the years, many options have been proposed and tested, including fiberglass, mineral wool, and foams (e.g., polyurethane, PU, and polyvinyl chloride, PVC) [3].

PU is formed of stiff and flexible segments, endowing PU foams with versatile properties and light weight and making them particularly desirable for insulation. They are obtained by a reaction between polyfunctional alcohols (polyol polyether or polyol polyester) and polyisocyanate [4]. Their foaming appearance is possible due to the production of a blowing agent (e.g., carbon dioxide) during exothermal polymerization, which remains enclosed within the material and ensures the foam insulating performance [5,6]. Depending on the amount, proportions, and characteristics of the components, three categories of PU foams can be defined: Flexible, semi-rigid, and rigid, the last being the preferred for insulation purposes due to its highly cross-linked and closed-cell structure, good mechanical and chemical resistance, low density, and low water adsorption [7]. Further, the R-value (measure of how well a two-dimensional barrier resists the conductive flow of heat) of rigid PU foams is among the highest of any insulating material, thus ensuring efficient heat retention and/or consistent temperature control of refrigerated environments [8].

In recent years, the development of PU-based composite foams with interdisciplinary functions has expanded considerably with the goal of increasing their mechanical performance, broadening their application, and preserving the environment by using lower amounts of PU [9,10]. Natural fibers have attracted much attention as potential reinforcements for composites due to their availability, biodegradability, and low cost [11]. These fibers have been involved in a growing type of polymer composites, the eco-composites, which describe combinations of materials with environmental and ecological potential and/or produced using materials from renewable resources [12,13,14]. So far, vegetable fibers such as flax, hemp, jute, and kenaf have been the most explored due to their low density, variable mechanical properties, and intrinsic biodegradability [15,16,17]. However, animal-based fibers are starting to demonstrate their potential as well. Feather keratin fibers have been shown to possess a hollow structure, filled with air, responsible for their low density and low dielectric constant, properties highly desirable in composites for electronic or automotive applications [18,19]. Silk fibers have been investigated to produce composites for tissue engineering due to their increased oxidation resistance and improved antibacterial and UV-light protection properties [20]. Animal-derived wastes, such as wool fibers, have also been successfully embedded in a polymeric film-forming matrix of cellulose acetate, with potential applications in the packaging and agricultural industries [21].

Even though this is an environmentally friendly solution to animal waste disposal, very few reports have been published on the subject.

In the present work, we explore the use of discarded dog wool fibers as a reinforcement agent in the production of PU-based eco-composites for thermal insulation. According to the Statistical Institute of Brazil (IBGE), there are in the country 52 million dog pets. The goal was to determine the efficiency of this mixture and the potentialities of animal wastes for industrial applications. To the authors’ knowledge, this is the first report on the use of dog wool fibers as reinforcement in PU-based eco-composites. Various fiber percentages were combined with PU castor oil. The resulting eco-composite foams were characterized in terms of their physical, thermal, and mechanical properties in light of the desirable application.

## 2. Materials and Methods

### 2.1. Materials

Respan, a semi-flexible and biodegradable polyurethane from castor oil (PU) resin, acquired from Resichem Chemicals LTDA (São Paulo, Brazil), was used as matrix. Castor oil is a vegetable oil pressed from castor beans. Dog wool fibers were used as reinforcement and were collected in pet shops in the metropolitan area of the city of Natal (Natal, Brazil). PU was used as control. All remainder chemicals were acquired from VWR International and used without further purification.

### 2.2. Treatment of Dog Wool Fibers

Dog wool fibers were initially washed in 0.05 M sodium hydroxide (NaOH) solution, to remove impurities present along the surface, and dried at 50 °C for 24 h. After, they were ground in a micro-slicer (Urschel, Chesterton, IN, USA) to obtain microparticles of ≈30 mesh screen.

### 2.3. Preparation of Eco-Composites

**Figure 1:**
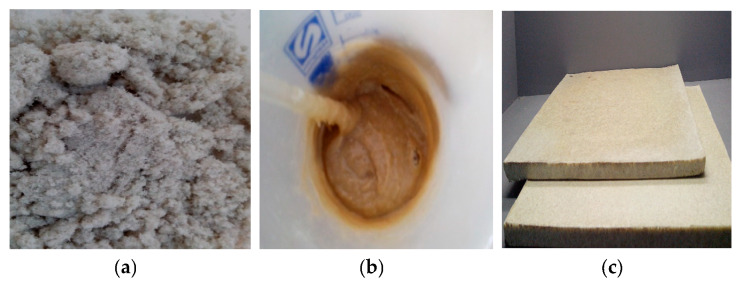
(**a**) Fiber microparticles, (**b**) mixture of PU resin and the fiber microparticles, and (**c**) the eco-composite.

Dog wool microparticles were thoroughly mixed with the semi-flexible PU resin using a commercial mixer, to guarantee the homogeneity of the composite structure. The mixture was then poured onto a steel mold, which was tightly closed, and submitted to a controlled expansion process to induce strong interactions between matrix and reinforcement (Figure 1). Eco-composite plates were produced with dimensions of 30 × 30 × 1 cm^3^ and different ratios of fiber in their composition (Table 1). Then, 100% PU plates were also produced and used as control.

### 2.4. Scanning Electron Microscopy (SEM)

Morphological analyses of the fibers and the eco-composites were carried out using a SEM TM 3000 HITACHI (Hitachi, Chiyoda, Tokyo, Japan). Backscattering electron images were realized with an acceleration voltage of 15 kV that enabled the visualization of the distribution of the fiber reinforcement along the polymeric matrix.

### 2.5. Particle Size Distribution

The particle size distribution was performed in a laser diffraction particle size analyzer model CILAS 1180 (Cilas, Orléans, France) at the laser light wavelength of 635 nm. The equipment is able to measure particles ranging from 0.04 to 2500 µm. The size distributions of the samples were determined based on Fraunhofer diffraction theory and expressed as frequency (%) vs. particle diameter (µm). The measurement was carried out with samples of 0.2 g in accordance to the standard BS ISO 13320:2009.

### 2.6. Fourier-Transformed Infrared (FTIR)

FTIR spectra of the eco-composites with various reinforcement percentages were collected using a Shimadzu spectrometer, model FTIR-8400S, IRAffinity-1 (Shimadzu, Kyoto, Japan), coupled with an attenuated total reflectance (ATR) accessory, the PIKE MIRacle™ single reflection with a ZnSe crystal (PIKE Technologies, Madison, WI, USA). Spectra were obtained in the range of 4000–500 cm^−1^, from 30 scans at a resolution of 4 cm^−1^. All measurements were performed in triplicate.

### 2.7. Thermal Properties

The thermal properties in the castor polyurethane samples were determined using the KD2 Pro (Decagon Devices, Pullman, WA, USA) equipment coupled with a thermal sensor twin needle SH1, which uses the transient line heat source method to measure thermal diffusivity, specific heat (heat capacity), thermal conductivity, and thermal resistivity. All analyses were performed at room temperature following the standards ISO EN 31092-1994. An average of 10 readings was taken for each sample and the data were reported as mean ± standard deviation. TGA was performed on a DTG-60H model (Shimadzu, Kyoto, Japan) using a platinum pan. The TGA trace was obtained in the range of 30–300 °C under nitrogen atmosphere, flow rate of 50 mL/min, and temperature rise of 10 °C/min. Results were plotted as percentage of mass loss vs. temperature. DSC was carried on a Power Compensation Diamond DSC (Perkin Elmer, Waltham, MA, USA) with an Intracooler ILP, based on the standards ISO 11357-1:2016, ISO 11357-2:1999, and ISO 11357-3:1999. Samples were dried at 60 °C for 1 h and placed in an aluminum sample pan before testing. The analysis was carried out in nitrogen atmosphere with a flow rate of 50 mL/min. The DSC analysis was carried out at three stages: The first heating, cooling, and second heating, all at the heating rate of 10 °C/min, in order to eliminate the thermal history of the samples. The thermogram was obtained in the range of 20 to 500 °C.

### 2.8. Mechanical Properties

The eco-composites’ tensile strength and compression capacities were examined using an X 300KN Universal Testing Machine (Shimadzu, Kyoto, Japan). The tensile strength of the eco-composites was determined following the ASTM D3039 with a specimen of 3 mm of thickness and 25 mm of width (75 mm^2^ of cross-section) and the compression test according to NBR 8082. In the compression test, deformation was measured when the machine was activated to reduce the thickness of the specimen in 10% at speed of 0.25 cm/min. It was calculated by the formula *R*_c_ = F/A, where *R*_c_ is the compression strength at 10% deformation (Pa), F is the force (N), and A is the test area of the sample (m^2^).

### 2.9. Hydration Capacity

The eco-composites’ water absorption capacity was measured following the ASTM D2842. Three replicates were used of each eco-composite. Samples were initially dried at 50 °C for 24 h and then transferred to a desiccator and left for 15 min until they reached room temperature. Samples were weighed in their dry state (mdry). After, they were immersed in distilled water (*d*H_2_O) and measured continuously (24 random intervals) until saturation was reached. The saturation point was determined when the sample weight reached a constant value (mwet). The samples’ hydration capacity was determined using the Equation (1):

(1) $$\%\;Water\;Absorbed=\frac{m_{wet}-m_{dry}}{m_{dry}}\times 100$$

### 2.10. Dilatometry

The thermal expansion coefficient of the samples was determined on the device NETZSCH model DIL 402 PC (Netzsch, Selb, Germany). The samples were made with dimensions of 25 mm in length and 8 mm in diameter. The tests were carried out under an argon gas flow of 5 mL/min at the heating gradient from room temperature to 170 °C. The heating rate was 5 °C/min.

## 3. Results and Discussion

### 3.1. Particle Size and Eco-Composites’ Morphology

**Figure 2:**
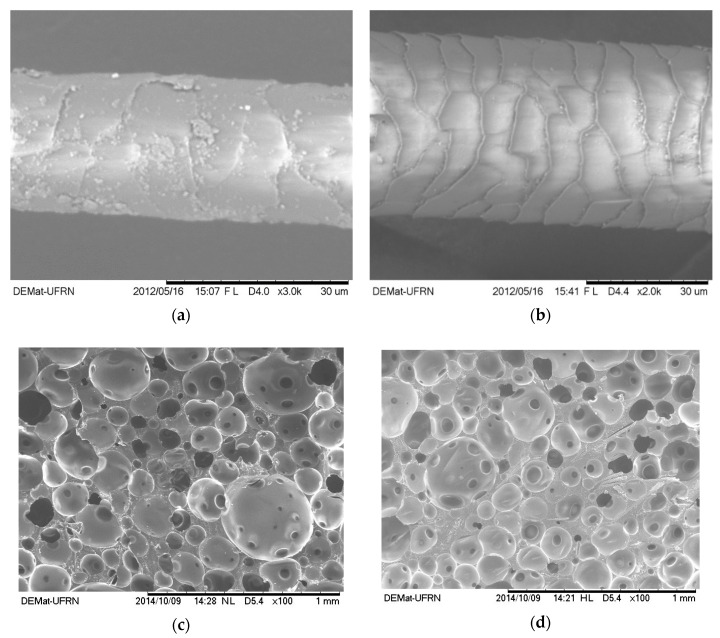

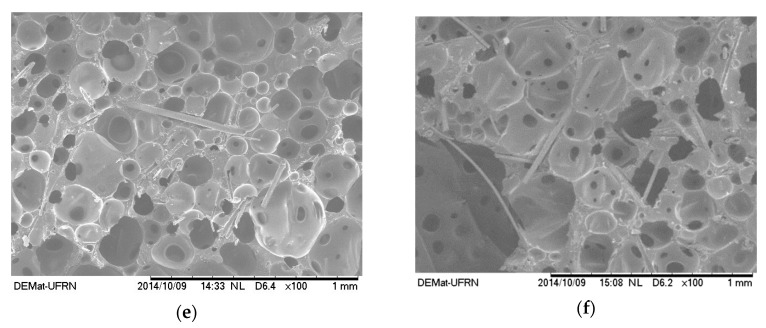
SEM micrographs of the dog wool fibers (**a**) in their natural state and (**b**) after treatment with 0.05 M of NaOH. Micrographs of the pure PU (**c**), PU + 10% of fibers (**d**), PU + 15% of fibers (**e**), and PU + 20% of fibers (**f**) at 100× magnification.

SEM micrographs of the dog wool fibers, in their natural state (untreated), treated with NaOH, and combined with the PU resin as reinforcement to form eco-composites were taken (Figure 2). As expected, there were substantial differences between the fibers before and after treatment with NaOH. The impurities present along the fibers (Figure 2a) were eliminated after NaOH washing, revealing the efficiency of this alkali treatment and leaving the surface clean and unspoiled (no evidences of degradation, Figure 2b), with a desirable open structure capable of interacting with the polymeric matrix. The incorporation of the fibers within the PU matrix was evidenced in Figure 2d. As can be observed, the porosity and morphology of the composite up to 15% of fiber content (Figure 2e) was not significantly different from the pure PU resin (Figure 2c). A very porous structure is characteristic of the PU foam. The average pore size (mean of 50 measures) of the composite was 84 ± 50 µm and the pore size of the pure PU was 83 ± 40 µm. At the fiber content of 20%, the PU structure became instable with large (~0.5 mm) collapsed structures and holes of ~0.2 mm around the fibers. Even though fibers’ distribution and orientation were random, they were preferentially detected in compact areas, both in the interior and the borders of the foam cells. Similar outcomes were obtained with composites of rigid PU foam reinforced with cellulose fiber residues [5].

**Figure 3:**
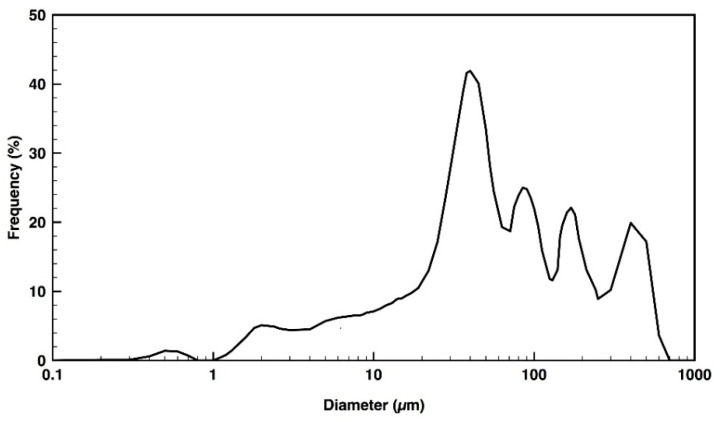
Dog wool microparticle size distribution.

Regarding their size, the dog wool fibers were considered microparticles. According to data from Figure 3, they were very heterogeneous in size, varying from 1 to 700 µm, with the largest amount measuring between 30 and 40 µm. This heterogeneity is to be expected since the dog wool wastes were collected from various pet shops that use different wool treatments and cutting tools. These factors can then condition the micro-slicer precision and, consequently, the grounding process.

### 3.2. ATR-FTIR Spectra

**Figure 4:**
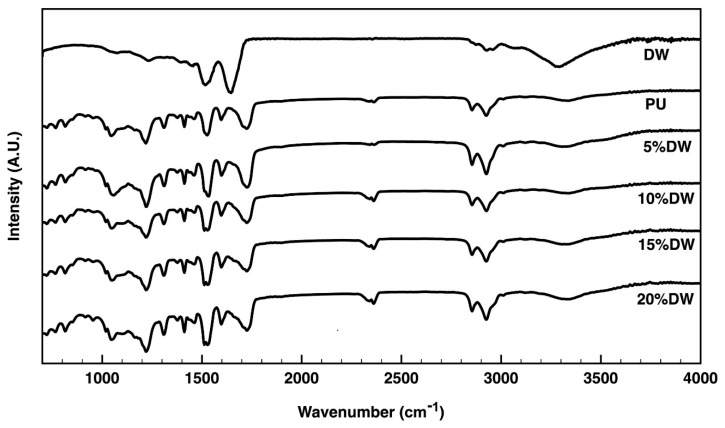
ATR-FTIR spectra of the pristine PU and Dog Wool (DW) and of the eco-composites containing 5% to 20% of dog wool microparticles.

The spectra profiles of the eco-composites formed of PU and different amounts of dog wool microparticles are shown in Figure 4. Between 3200–3450 cm^−1^ was located one of the most important PU bands. This was attributed to the symmetric and asymmetric stretching vibrations of the N-H groups from the urethane and urea, which result from the reaction between water and isocyanate [22]. However, as observed by the spectrum of the dog wool fibers, a large peak at 3300 cm^−1^ is typical of the stretching vibrations of –NH groups in keratin [23]. As the amount of dog wool fibers increased in the composite, this region became broader, which indicates a larger number of intermolecular hydrogen being promoted by these microparticles [24]. A very small peak corresponding to C–H stretching of the aliphatic CH=CH was identified at 3008 cm^−1^, while at 2950 and 2850 cm^−1^ the asymmetric and symmetric stretching vibrations of C–H were observed, respectively. A peak around 2270 cm^−1^, associated with the stretching vibrations of the NCO group of the isocyanate, was detected in all formulations. However, it was more important in those eco-composites containing higher amounts of dog wool microparticles. This is indicative of the presence of unreacted isocyanate [25]. The peaks at 1710, 1240, and 1070 cm^−1^ relate to the stretching vibrations of the C=O and C–O of the ester groups, while the overlapping bands between 1540 and 1517 cm^−1^ can be attributed to the stretching and bending vibrations of the C–N and N–H of the urethane moieties, respectively. These two peaks could also be assigned to the C–N stretching and N-H bending vibrations of amide II in wool fibers. This explains the increasing definition and clarity of these two peaks as the percentage of dog wool fibers rose in the eco-composite [26]. A very small increase in the composite of the peak at 1650 cm^−1^ can be observed. This peak in the dog wool spectrum is attributed to the α-helix of the keratin structure [27]. This peak can be considered as a direct measure of the presence of the fiber in the composite.

### 3.3. Thermal Properties

**Figure 5:**
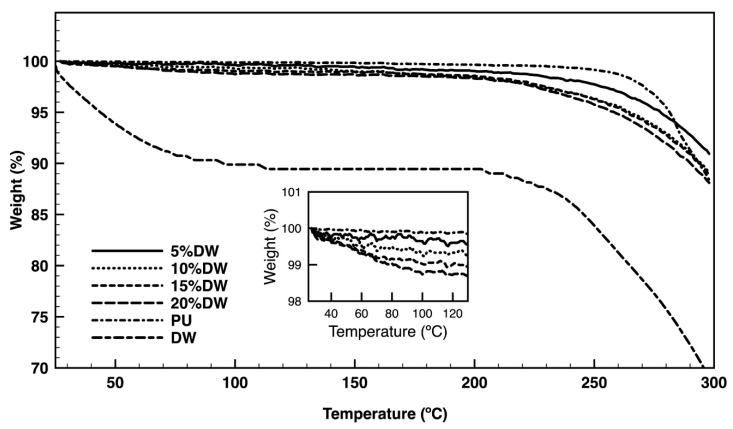
TGA of pristine PU and dog wool (DW) and the eco-composites containing 5, 10, 15, and 20% of dog wool microparticles measured between 25 and 300 °C, performed at a heating rate of 10 °C/min in a nitrogen atmosphere. The inset represents the initial part of the PU and PU composites between 25 and 130 °C.

Degradation steps associated with temperature rising were identified on PU and PU-based eco-composites via TGA (Figure 5). In the pure dog wool, a weight loss between 25 and 100 °C was observed due to the evaporation of the incorporated water. The second decomposition starting at around 200 °C could be attributed to the denaturation and degradation of the keratin molecules. According to literature, the disulfide bonds are cleaved between 230 and 250 °C [28]. In the composite, but not in the pure PU, a first very small step of degradation was detected between 25 and 100 °C (Figure 5 inset) and refers to the initial volatilization of moisture from the foams due to the evaporation or dehydration of hydrated cations [29,30]. This step was more important on the fiber-reinforced composites because of the wool fibers’ affinity towards water molecules, which tends to increase moisture retention [24,31]. The first degradation step for the pristine PU was detected at ≈260 °C and was attributed to the cleavage of the PU polymeric backbone, initiating with the polyol component degradation (urethane chains) and, then, progressing to the isocyanate component degradation (ester bonds) [32]. At 300 °C, 12% of the original mass was already lost with the remaining 88% being further decomposed into amines, small transition components, and CO_2_ [33]. Because of the wool fibers’ incorporation, the eco-composites were more quickly prone to degradation. This occurred because keratin wool fibers, such as dog hair fibers, start decomposing at temperatures superior to 200 °C. In fact, from this point, denaturation of the helix structure and the destruction of chain linkages, peptide bridges, and the skeletal degradation occurs. At temperatures closer to 300 °C, several chemical reactions take place with the fibers being decomposed into lighter products and volatile compounds such as CO_2_, H_2_S, H_2_O, and HCN [34]. From all formulations, the eco-composites containing 5% of dog wool microparticles were capable of retaining more of their original mass, ≈ 91%, at 300 °C.

**Figure 6:**
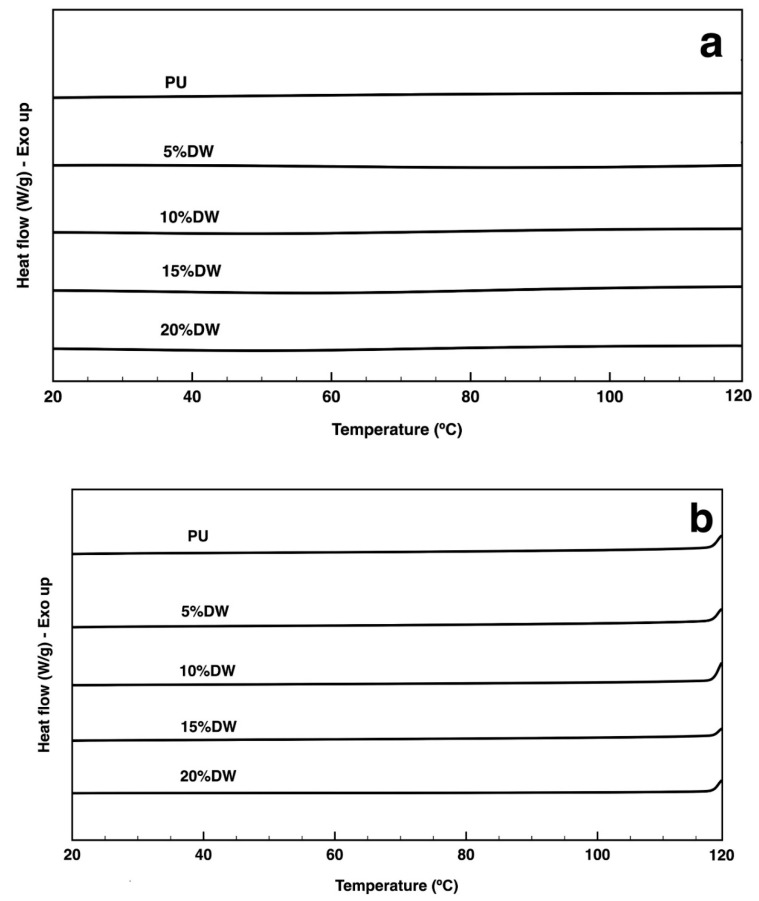

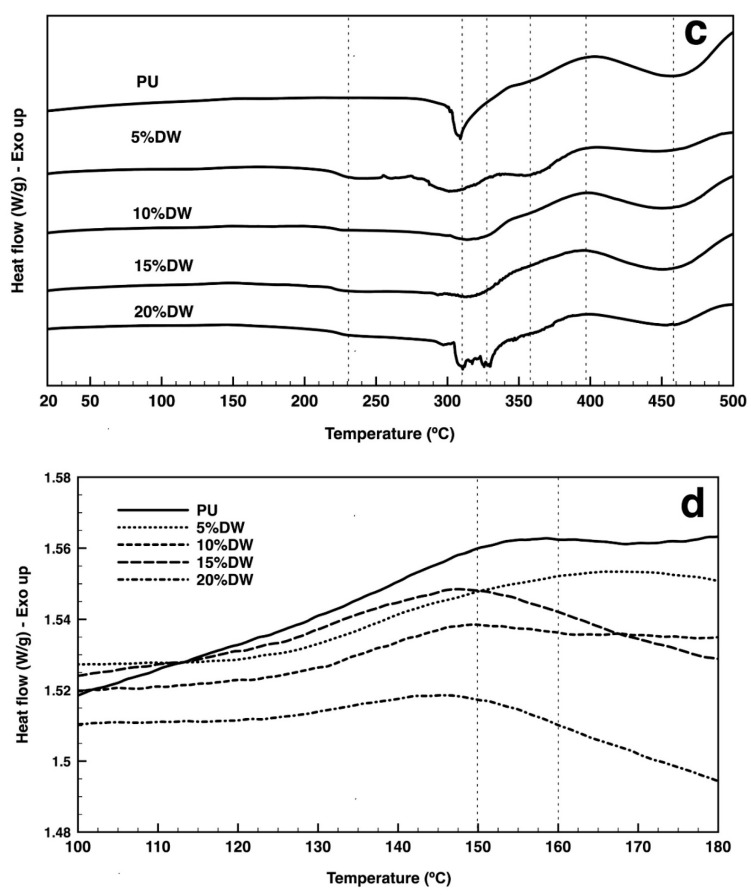
DSC thermogram of the pristine PU and the eco-composites containing 5, 10, 15, and 20% of dog wool microparticles collected at a heating rate of 10 °C/min in a nitrogen atmosphere. (**a**) The first heating cycle between 20 and 120 °C, (**b**) the first cooling cycle between 120 and 20 °C, (**c**) the second heating cycle between 20 and 500 °C. (**d**) Detail of the second heating cycle between 100 and 180 °C showing the *T*_g_.

DSC thermograms of the PU and PU-based composites prepared with different percentages of dog wool microparticles were acquired between 20 and 500 °C (Figure 6). The first heating cycle between 20 and 120 °C (Figure 6a) and the cooling cycle between 120 and 20 °C (Figure 6b) did not shown any significant event. In the second heating cycle (Figure 6c), the first endothermic peak for PU was detected at ≈300 °C, which, as seen earlier, is associated with the cleavage of the PU polymeric backbone, initiating with urethane chains and continuing to the ester bonds. In Figure 6d, it can be observed a detail of the second heat cycle between 100 and 180 °C. In this region a *T*_g_ is observed in all the thermograms relative to the hard urethane segments [35]. However, the *T*_g_ of the composites starting from 10% of dog wool content were lower (~150 °C) than corresponding PU control (~160 °C) and the 5% composite. It seems that the presence of the fibers affected the state of crystallinity in the PU matrix by reducing the *T*_g_ towards lower temperatures. These *T*_g_ are very small since polyurethane is mostly amorphous and suggest that the fibers improve the mobility of soft segment in PU, reducing the hydrogen bonding interactions [36]. For the eco-composite foams, the first endothermic peak occurred earlier, at ≈220 °C, with the initial denaturation of the wool fiber helix structure and the destruction of chain linkages. At temperatures ranging from 300 to 340 °C, the main polymeric chains in the eco-composite started degrading together with the remaining components of the wool fibers. These data are consistent with the TGA observations. The last endotherm peak registered for all foams was detected around 460 °C and can be attributed to the final degradation of the remaining residual polymeric chains and dog wool fibers into carbon char, small transition components, and volatile species [33,34].

In foamed systems, the dominant heat transfer modes are thermal radiation and gas-gas and solid-solid conduction. In PU foams, the total conductivity ranges about two-thirds of the conductivity of stagnant air because there is low conductivity gas, or foaming agent, inside the foam [33]. Here, the addition of the wool fibers to the eco-composites had little influence on the foams’ thermal conductivity (Table 2), maintaining the values within the expected ranges, desirable for thermal insulation, and approximated to those of polystyrene (one of the most common materials applied in thermal insulation) [37,38,39]. The thermal or heat capacity measures the amount of energy required to raise the temperature of a material one degree. Data from Table 2 demonstrates, again, that the PU and the eco-composites presented very similar values. However, the addition of 20% dog wool microparticles increased the composite thermal capacity above the pristine PU. Hence, this formulation requires more heat for temperature variations to occur, thus maintaining insulation more effectively. Thermal diffusivity describes the rate of temperature spread through a material and is a function of the thermal conductivity and the heat thermal capacity. As such, since thermal conductivity was the lowest in the eco-composites containing 20% of dog wool fibers, the same happened with the thermal diffusivity. It has been shown that thermal diffusivity is dependent on the organization of the foaming cells, their dimension, and the type of blowing agent applied [40]. Here, it is likely that the random disposition of the microparticles along the polymeric matrix may have compromised these specific thermal properties. Finally, in order to be classified as an insulating material, the foam must be endowed with a high thermal resistance. Data shows that thermal resistance decreased slightly with the addition of dog wool fibers. Even though these values are acceptable for thermal insulation, it seems that by increasing the percentage of fibers within the eco-composite this property is also enhanced. Thus, future studies will be conducted to confirm this premise.

### 3.4. Mechanical Properties

**Figure 7:**
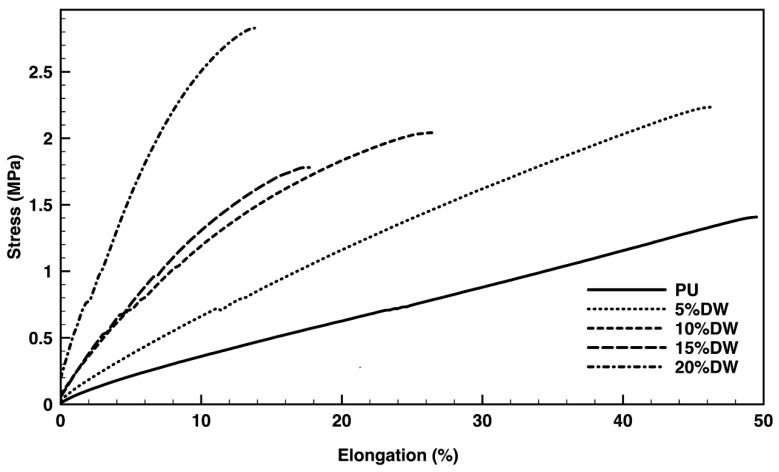
Stress (MPa) versus elongation at break (%) of the pristine PU and the dog wool-reinforced eco-composites.

**Figure 8:**
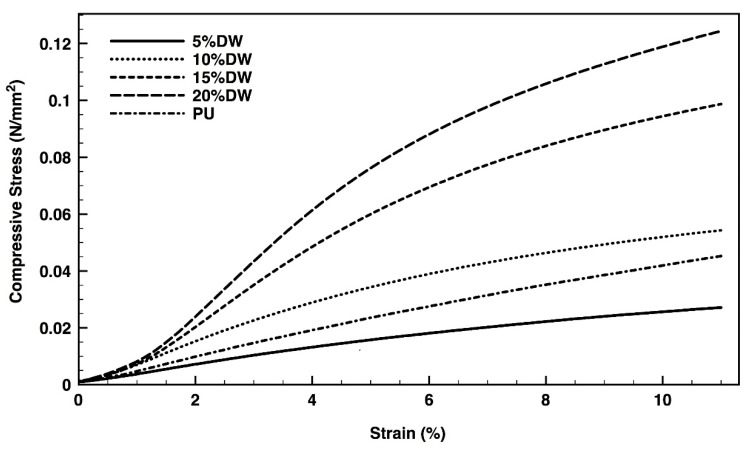
Compressive stress versus strain of the pristine PU and the dog wool-reinforced eco-composites.

The foams’ tensile stress and compression performance were measured with and without the addition of the dog wool microparticles (Figure 7 and Figure 8, respectively). PU achieved the highest percentage of elongation from the tested formulations (≈50%), although requiring less stress (≈1.25 MPa) to break than the reminding eco-composites. In fact, with the addition of only 5% dog wool microparticles, the stress necessary to reach a similar elongation state (≈46%) was almost double, ≈2 MPa (Figure 7). This behavior is explained by the interactions established between the polymer matrix and the fiber arrays, which led to the disorganization of the PU original structure. At this percentage, small changes were induced in the foam’s morphology. It is possible the microparticles migrated and filled existing defects, thus increasing the force necessary to break the material. A stiffness enhancement was registered with superior percentages of dog wool fibers. This is related to the higher rigidity of the foam solid phase in consequence of the fiber’s contribution [5]. Because of the heterogeneous and disorganized orientation and distribution of the fibers along the composite, there was no proportion between the force applied-elongation capacity and the percentage of fiber reinforcement.

Even though the 5% eco-composites registered the most balanced performance between stress applied and elongation capacity (Figure 7), their resistance to compression was the lowest from the group (Figure 8). It is likely the rearrangements the polymeric foam underwent, to accommodate the fibers, promoted the development of an anisotropic-like material in which the mechanical resistance was more important in one direction than in the other [41]. Also, the presence of gaps along the foam in response to the addition of the microparticles and the alterations in the PU original structure may have contributed to this phenomenon. Irregularities in the foams’ organization are more likely to occur in composites containing smaller amounts of reinforcement fibers than higher [5]. In turn, the increased percentage of filler can induce a decrease in the reactivity of the components in the system, affecting the foam expansion and increasing its density and rigidity, consequently, improving the compression strength [42]. As such, it was expected the maximum compressive stress to strain to be endured by the composite with the largest percentage of filler (20%).

### 3.5. Hydration Capacity

**Figure 9:**
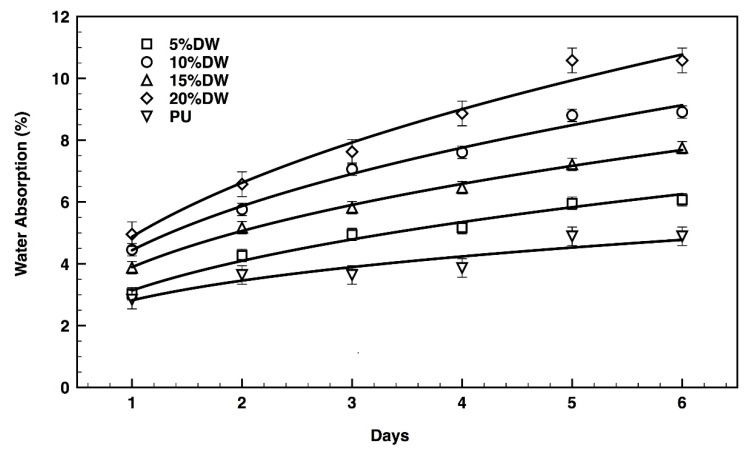
Water adsorption capacity of the pristine PU and the dog wool-reinforced eco-composites over time.

The water adsorption capacity of the pristine PU and the dog wool-reinforced eco-composites was followed up to six days in *d*H_2_O, with samples being weighed every 24 h until water saturation was reached. Data from Figure 9 revealed pristine PU as the foaming material with the least hydration capacity, reaching a saturation state with only 4% of water in its composition. The eco-composites were found more attractive to water molecules with their hygroscopic capacity augmenting as the percentage of microparticles increased, that is, from 5% water content in the 5% dog wool eco-composites to 11% water content registered for the 20% dog wool-reinforced eco-composite. These results are explained by the ability of wool fibers to bind and absorb large amounts of water [31]. Water permeability in wool fibers is dictated mainly by cell membrane lipids. However, much remains to be understood on this front. The interaction between fibers and water is quite complicated; at low relative humidity, a water molecule monolayer can be formed by the interaction with specific fiber polar side chains, while at high relative humidity water associates with the peptide backbone of the fiber, generating a multilayer absorption. Fiber swelling also occurs, as a result of the breaking of hydrogen bonds between and within protein chains, due to water molecules rising over the surface and within the intercellular spaces; thus, generating even more interaction sites for water molecules [43,44].

### 3.6. Dilatometry

**Figure 10:**
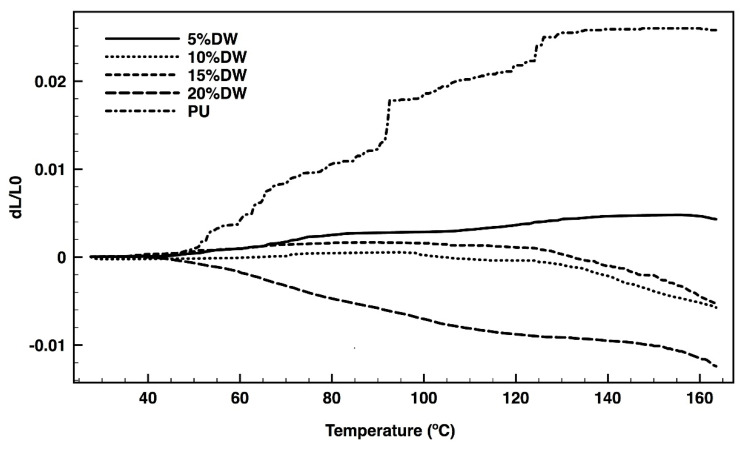
PU and eco-composites’ dilatation with increasing temperature, from 30 to 160 °C.

Thermal expansion is defined as the increase in a material’s volume in response to temperature rising. As the temperature rises, molecular agitation increases thereby growing the distance between the molecules. Figure 10 shows evidences of PU dilation with the increase in temperature from 50 to 160 °C. The addition of the dog wool fibers reduced significantly the polymer expansion with temperature variations. In fact, with a 5% addition of microparticles, the foam’s volume did not even alter. By incorporating a higher content of wool fibers, the foam’s dilatation reached negative values, indicative of the loss in material stability and capacity to maintain its structural integrity with heating. The best results showing structural stability up to 120 °C were obtained using a maximum of 15% of dog wool fibers.

## 4. Conclusions

PU eco-composites reinforced with dog wool fibers were successfully produced at different percentages. Alkaline treatment was effective in removing impurities from the fibers without compromising their integrity. Fibers were incorporated along the polymeric matrix in a random and disorganized manner. Still, they were effective in increasing the foams’ mechanical resistance, namely tensile and compression strengths. The thermodynamic behavior suffered little changes with the incorporation of the dog wool fillers, being the most important the improvement in thermal capacity. Additionally, the hydration capacity was significantly improved in response to the wool fibers’ water permeability and increased capacity to bind with water molecules. Dilatometry studies revealed the capacity of the fillers to increase the thermal stability of the foam, reducing their expansion with heating. Data demonstrated the potential of this combination to produce new alternative solutions for insulation using low-cost, sustainable resources and with minimal environmental impact.

## Figures and Tables

**Table 1:** Eco-composites’ composition.

Eco-Composite (%)	Dog Wool (g)	PU (g)
**5**	12.5	237.5
**10**	25.0	225.0
**15**	37.5	212.5
**20**	50.0	200.0

**Table 2:** Main thermal properties of pristine PU and the eco-composites (*n* = 3, S.D. ± 3).

Samples	Thermal Conductivity (W/mk)	Thermal Capacity (MJ/m^3^k)	Thermal Diffusivity (m^2^/s)	Thermal Resistance (°C cm/W)
**PU**	0.053 ± 0.004	0.561 ± 0.045	0.091 ± 0.003	1878.5 ± 153.3
**5% DW**	0.064 ± 0.006	0.454 ± 0.015	0.141 ± 0.016	1576.0 ± 153.3
**10% DW**	0.070 ± 0.002	0.603 ± 0.048	0.122 ± 0.006	1411.5 ± 61.7
**15% DW**	0.063 ± 0.002	0.530 ± 0.046	0.120 ± 0.009	1590.0 ± 38.2
**20% DW**	0.061 ± 0.002	0.615 ± 0.053	0.098 ± 0.012	1647.5 ± 45.4
